# Evolution of WIfI: Expansion of WIfI Notation After Intervention

**DOI:** 10.1177/15347346221122860

**Published:** 2022-09-01

**Authors:** Virginie Blanchette, Malindu E. Fernando, Laura Shin, Vincent L. Rowe, Kenneth R. Ziegler, David G. Armstrong

**Affiliations:** 1 Southwestern Academic Limb Salvage Alliance (SALSA), Department of Surgery, 12223 Keck School of Medicine of University of Southern California, Los Angeles, CA, USA; 2 Department of Human Kinetics and Podiatric Medicine, 14847 Université du Québec à Trois-Rivières, Trois-Rivières, Québec, Canada; 3 Ulcer and wound Healing consortium (UHEAL), Queensland Research Centre for Peripheral Vascular Disease, College of Medicine and Dentistry, 104560 James Cook University, Townsville, Queensland, Australia; 4 Faculty of Health and Medicine, School of Health Sciences, University of Newcastle, Australia

**Keywords:** diabetes, foot ulcer, wound healing, peripheral vascular disease, ischemia, classification, amputation

## Abstract

Nearly a decade ago, the Society for Vascular Surgery (SVS)'s wound, ischemia, and foot Infection (WIfI) classification was first developed to help assess overall limb threat. However, managing conditions such as diabetic foot ulcer and chronic limb-threatening ischemia can be complex. For instance, certain investigative findings might initially be pending such as the level of ischemia or extent of infection before the final classification is established. In addition, wounds evolve rapidly, and the current classification does not allow for tracking their progression over time during treatment. Therefore, we propose a supplemental consistent notation for scoring WifI re-assessment during treatment of a threatened limb inspired by the cancer staging before and after neoadjuvant treatment classification system. Thus, we describe the re-scoring system and how to use it. Our suggestion supports a coherent method to longitudinally communicate characteristics of a threatened limb. This has potential to support high quality interdisciplinary, patient-centered care and enhance the use of this classification in research. Further work is required to validate this modification of a common language of risk.

Diabetes-related foot complications such as diabetic foot ulcers (DFUs), peripheral arterial disease including critical limb-threatening ischemia (CLTI), Charcot neuro-arthropathy and lower extremity amputations (LEAs) are a leading cause of global morbidity, mortality, reduced quality of life and direct and indirect healthcare costs.^1–3^ Indeed, 5-year mortality rates associated with these complications are greater compared to many cancers.^4^ Limb complications of diabetes increase in people with multimorbidity such as concomitant nephropathy and cardiovascular disease.^4–6^ People with an history of DFUs are at increased risk of ulcer recurrence.^1^ In fact, 40% of those people will develop DFU recurrence within a year, 65% within 5 years, and greater than 90% within 10 years.^1,7^ Because the epidemiology of diabetes-related foot complications is comparable to that of cancer, and recurrence is common, after the initial healing of an index DFU, it is appropriate to refer to a person not as cured of DFU, but rather as being in “DFU remission”.^8^ In addition, as with cancer, the complexities associated with management require a comprehensive and organized team approach, including the patient, their family and caregivers, to achieve the best outcomes and high quality patient-centered care.^9–12^

Nearly a decade ago, Mills, Conte, Armstrong and coworkers, in concert with the Society for Vascular Surgery (SVS) proposed an integrated lower extremity wound classification system related to LEA risk and revascularization benefit.^13^ Inspired by cancer research, it is intended to define the disease burden, analogous to the tumor, node, metastasis (TNM) system for cancer staging.^13,14^ WIfI consists of a graded scoring system for wound, ischemia, and foot infection. For any given threatened limb, a severity grade of 0 to 3 (ie, none, mild, moderate, severe) is assigned to grade the severity and extent of wound, ischemia, and foot infection, respectively (Figure 1). On the basis of these three scores, patients are further assigned to four threatened limb clinical stages corresponding to estimated risk of LEA derived by an expert panel consensus. The underlying premise of WIfI is that the risk of LEA increases as the presenting disease burden progresses from clinical stage 1 (very low risk) to stage 4 (high risk).^13^ However, managing the threatened limb can be complex, and the impact of conservative, surgical, and medical interventions on the stabilization of DFUs is not reflected in the scoring system. Additionally, certain investigative findings might initially be pending such as the level of ischemia or extent of infection before the final classification is established. DFUs evolve rapidly, and the current classification does not allow for tracking limb progression over time during treatment and related to potential recurrence of the index DFU. Moreover, these limitations create a logistical challenge for utilizing WIfI for large scale reporting including in cohort studies and clinical trials. Therefore, consistency in notation is required for widespread application.

**Figure 1.**
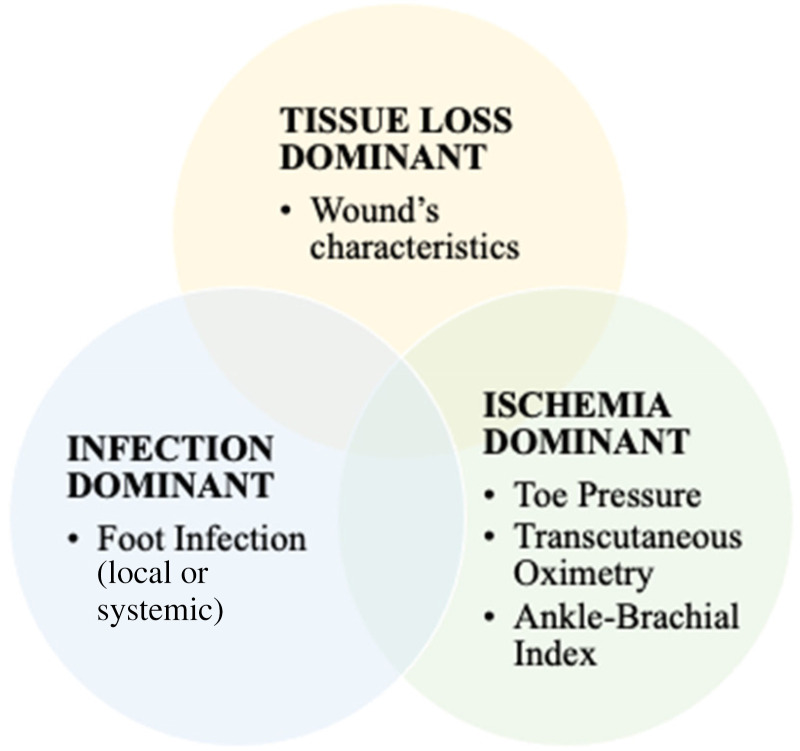
SVS WIfI classification system, adapted from Armstrong & Mills (2013).^15^

To continue the analogy of DFU with cancer staging, a recent publication suggested an updated classification to better reflect the situation after neoadjuvant therapies and to highlight the differences between clinical and pathological states.^16^ Given similar issues in categorizing DFUs post treatment such as revascularization and limb salvage, treatment of osteomyelitis or debridement or minor amputation, with a supplemental WIfI notation is appropriate and would assist the clinician as well as provide a consistent approach for use in clinical studies. A recent study demonstrated that WIfI restaging is an important tool for predicting limb loss and assessing adequacy of intervention, more so than baseline WIfI alone.^17^ Therefore, the aim of this communication is to propose a revised, consistent notation for scoring WIfI re-assessment during treatment of a threatened limb for the clinical and research community with the intention to make it practical to use on a daily basis. Thus, we describe the re-scoring system.

## Notation for re-Scoring WifI

In order to address the issue of either upgrading or downgrading the WIfI score of a threatened limb, and to reflect the actual condition, and to allow the latitude of a pending score, identifying the timeline to the event and associated interventions is important. Thus, it will be possible to classify a DFU for example, according to the stage of management: before treatment, during/after treatment and at remission. This is separated in four phases: primary presentation (p), during therapy (y), healed (h) or recurrent (r).

**Figure 2.**
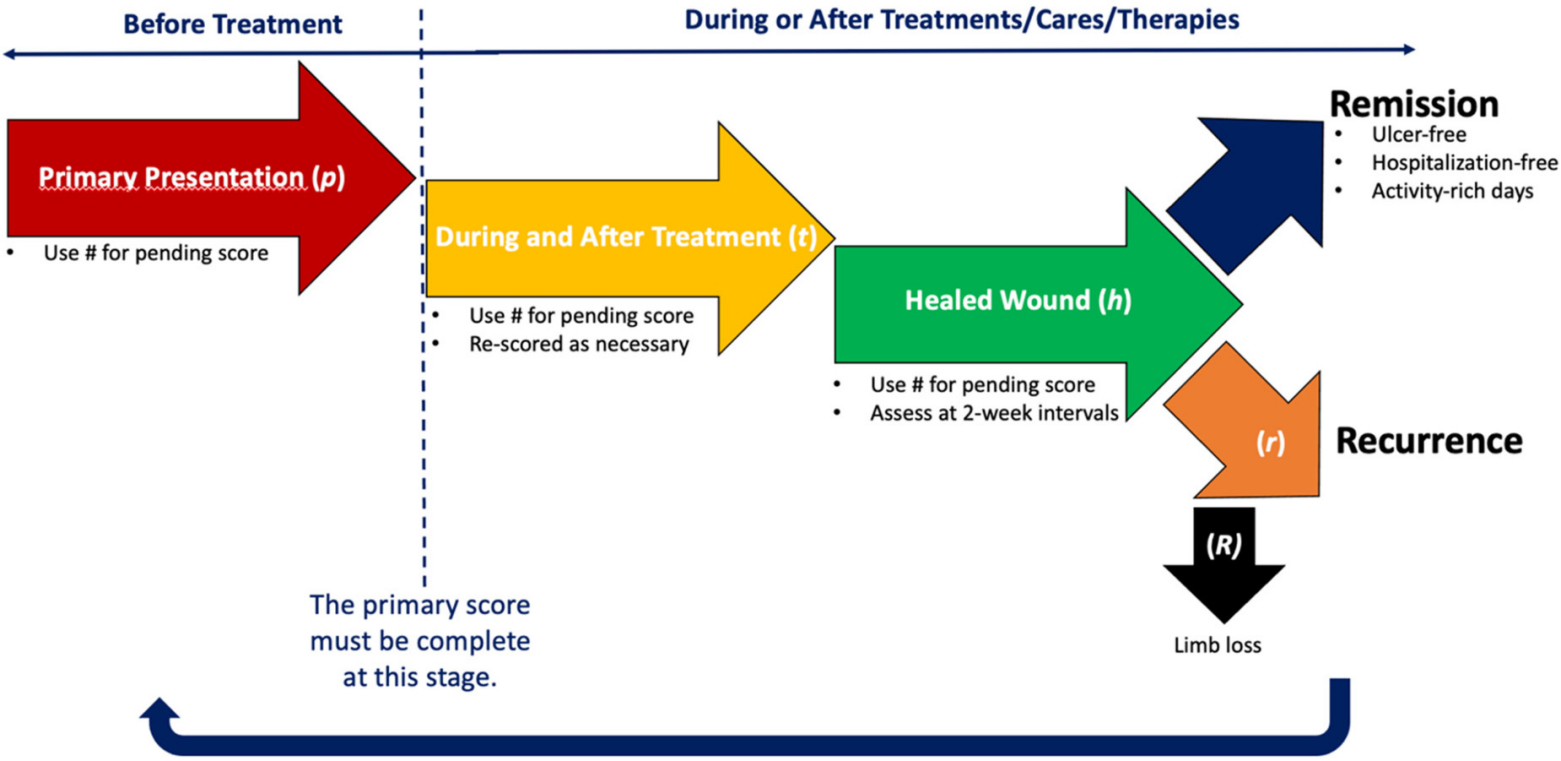
Schematization of the supplemental WIfI notation.

When there is missing data to complete the final WIfI score, for example, when the clinician is awaiting the results of a pending vascular investigation to identify the ischemia status, or awaiting the results of histology/laboratory data to support the presence of bone involvement in infection, the number is temporarily replaced by the hashtag (#) until the score can be completed with certainty. In this case, it is still necessary to identify the index phase (Figure 2).

Thus, for an index ulcer, the assessor (ie, clinician or researcher) should always establish a score for the primary presentation that is associated with the risk of major LEA and/or the benefits of revascularization as a preliminary WIfI score or “pWIfI” score. During and following the treatment, whatever the nature and number of treatments and the length of follow up, the scorer can define the classification as many times as needed by using a post treatment WIfI score or “tWIfI” score. Considering that the goal is to stabilize (or to maintain in remission) the threatened limb condition, it is relevant to repeat the score when the condition is deemed healed (h). The repeated score is still relevant as there could still be an ischemic component to follow. Therefore, the user should note the status using “hWIfI”.

A threatened limb, or healed limb is often evolving gradually, changing rapidly and is at high-risk of recurrence. Indeed, the healed WIfI score is not a static measurement and that it is in fact a cycle.

**Figure 3.**
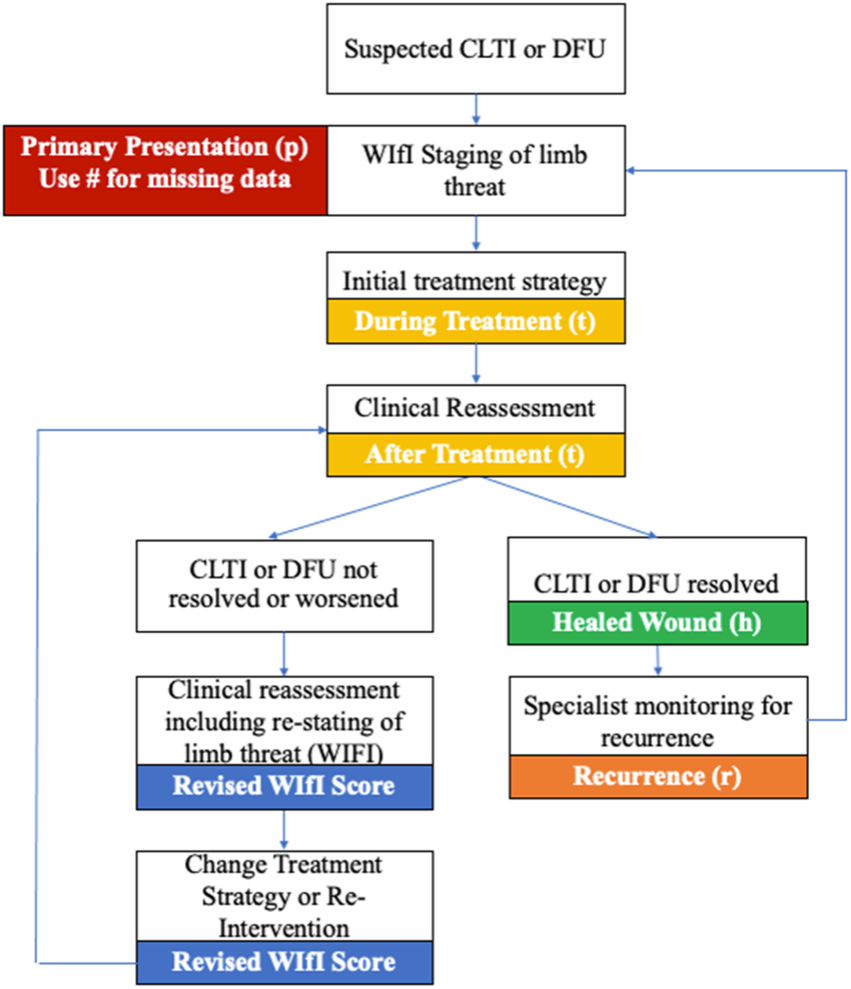
Continuous evaluation and re-staging. Adapted clinical algorithm from Conte and al. 2020.^20^ Abbreviation: WIfI: Wounds, Ischemia and foot Infection Classification; CLTI: Chronic Limb-Threatening Ischemia; DFU: Diabetic Foot Ulcer.

This cycle can then be restarted using the notation and this is based on the adapted clinical algorithm suggested by Conte and al. (2020) to implement limb staging with the WIfI as a part of the initial assessment of CLTI (Figure 3).^18^ On the other hand, the possibility that the cycle stops after the healing score is plausible. This indicates that the team has accomplished its mission to maximize ulcer-free, hospital-free, and activity-rich days, the same way a cancer survivor is deemed to maximize cancer-free days. ^4,19^ Alternatively, if limb salvage is not attained and limb loss ensues, then this is designated with a capital R to indicate limb loss due to an index ulcer as “RWIfI” This is a comprehensive re-notation that can be preconize in research context.

## Definitions

### Primary Presentation (p)

This is the first WIfI score that is assigned when the diagnosis of the limb-threatening condition such DFU and CLTI. This score is made according to clinical criteria established by former guidelines. The pending score should be completed before proceeding to the next phase considering that it is the score that determines the LEA risk and revascularization risk according to the initial WIfI.^13^ This represents the initial state without treatment. This should be evaluated by a trained and competent health care provider that can diagnose the components of wound, ischaemia and infection within their scope of practice.

### During Treatment/Therapy (t)

For our purpose, therapies, treatments, and cares are synonym to attempted remediation of the limb-threatening condition following the diagnosis. There are a multitude of treatments for the conditions that can supplement the WIfI score. Therefore, any action or way of treating a patient or a condition medically or surgically such as management and care to prevent, cure, ameliorate, or slow progression of a threatened limb condition is included in this phase. These treatments must be supported by the suitable guidelines and respect the ethical principles of research with human subjects. This score can be use in the initial treatment strategy using the holistic patient, limb, anatomy (PLAN) framework or during the clinical reassessment and can be re-scored according to the clinical reassessment as needed.

### Healed (or Controlled or Stable) (h)

Food and Drug Administration (FDA) defines healing as the 100% re-epithelialization of the wound surface with no discernable exudate and without drainage or dressing, confirmed at two visits two weeks apart.^21^ Moreover, a blinded adjudication for wound assessment is suggested in the case of a clinical trial.^22^ As discussed above, a DFU would be in remission instead of healed. The International working group on Diabetic Foot (IWGDF) defines it as an intact skin and absence of infection of the complete foot after healing of any DFUs.^23^ Thus, the final score associated with this phase should be established according to the above conditions, confirmed at two week intervals by a final blinded wound assessment.^24^

### Recurrence (r)

The IWGDF defines DFU recurrence as a new foot ulcer in a person who has a history of DFU, irrespective of location and time, since previous DFU.^23^ Nevertheless, with this notation, the recurrences are counted only if it is the same indexed DFU/wounds. If it is a new wound location, it is not a recurrence. This is supporting the complexity of the lower limb vascularization related to the healing potential and WIfI score.^25^ For a new wound, it is then necessary to established the score based on the primary presentation notation.

### Limb Loss (R)

Limb loss is any major amputation defined as any resection proximal to the ankle.^23^

## Strengths and Limitation

This consistent notation for re-scoring tissue loss, level of ischemia, and severity of foot infection assessment during treatment aims to meet the needs of both the clinical and scientific community to enhance person-centered care and inform better practices. Moreover, it has the potential to promote better understanding, communication, and follow-up within the team but also in broader dissemination In addition, it facilitates the use of the WIfI classification in a general way as we have established clear definitions for each phase according to the stage of clinical management. This addition does not distort the original SVS classification but can supplement the use of WIfI as needed. In the same way, it allows longitudinal evaluation of the person with wounds in relation with treatments (ie, before and after) including the number of recurrences if necessary. This notation is flexible considering the use of a specific notation (#) while waiting for new data from investigations as an example. The “pending” result was previously ambiguous. As the initial WIfI score was intended to assess 1-year major amputation risk or benefit of revascularization, our suggestion evaluates progression rather than assessing risk or benefit. Thus, these predictive values can be considered only when assessing the WIfI score of the primary presentation (p) or at the initial assessment for a recurrence (r). Considering the variability of treatment options and the great heterogeneity in terms of dose, exposure, time, number, follow up, etc, attributable to this stage of management, a general predictive value seems unrealistic. However, it can open the way to evaluate predictive values of a specific care pathway or treatments, and this 2.0 WIfI notation can accompany efforts in this direction. Lastly, the revised scoring system we have suggested requires validation and evaluation for repeatability, which we plan to undertake. It is consistent with previous work implementing global guidelines for CLTI in clinical practice.^20^

## Conclusion

Our suggestion supports a coherent way to longitudinally communicate characteristics of a threatened limb. This has potential to support high quality interdisciplinary patient-centered care. This is one more improved way in the “toolbox” of clinicians and researchers to maximize ulcer-free, hospitalization-free, activity-rich days for people with limb-threatening conditions, in the same way the team and cancer survivors strive to maximize cancer-free days. We look forward to further efforts to validate this modification of a common language of risk.
